# Primary-care observational database study of the efficacy of GLP-1 receptor agonists and insulin in the UK

**DOI:** 10.1111/dme.12137

**Published:** 2013-05-19

**Authors:** G C Hall, A D McMahon, M-P Dain, E Wang, P D Home

**Affiliations:** 1 Grimsdyke HouseLondon, UK; 2 University of Glasgow Dental SchoolGlasgow, UK; 3 Diabetes–Metabolism FranchiseSanofi-Aventis, Paris, France; 4 Diabetes–Metabolism FranchiseSanofi-Aventis, Bridgewater, NJ, USA; 5 Newcastle UniversityNewcastle upon Tyne, UK

## Abstract

**Aims:**

We investigated use and efficacy of glucagon-like peptide-1 (GLP-1) receptor agonists in UK practice.

**Methods:**

People starting a GLP-1 receptor agonist (exenatide, liraglutide) or insulin (glargine, detemir, NPH) after a regimen of two or three oral glucose-lowering agents were identified from The Health Information Network observational primary care database (2007–2011). Mean change in HbA_1c_ and body weight were compared at 1 year between cohorts, adjusting for baseline characteristics.

**Results:**

Baseline characteristics of GLP-1 receptor agonist (*n* = 1123) vs. insulin (*n* = 1842) users were HbA_1c_ 78 vs. 84 mmol/mol (9.3 vs. 9.8%) and BMI 38.2 vs. 30.9 kg/m^2^. The GLP-1 receptor agonist cohort was younger, had shorter diabetes duration and follow-up, less microvascular disease and heart failure, higher estimated glomerular filtration rate and more use of oral glucose-lowering agents. Lower HbA_1c_ reduction on GLP-1 receptor agonist [7 vs. 13 mmol/mol (0.6 vs. 1.2%) (*n* = 366 vs. 892)] was not statistically significant [adjusted mean difference −1.4 (95% CI −4.1, 1.2) mmol/mol], except in the highest HbA_1c_ quintile [>96 mmol/mol (>10.9%); adjusted mean difference −17.8 (−28.6, −7.0) mmol/mol]. GLP-1 receptor agonist users lost weight [−4.5 vs. +1.5 kg; adjusted mean difference 4.7 (3.7, 5.8) kg; *n* = 335 vs. 634]. A UK 6-month target reduction for GLP-1 receptor agonists of 11 mmol/mol (1.0%) HbA_1c_ and 3% weight was reached by 24.9% of those continuing treatment.

**Conclusions:**

Those starting GLP-1 receptor agonists are heavier with better glycaemic control than those starting basal insulin. Subsequently, they have improved weight change, with similar HbA_1c_ reduction unless baseline HbA_1c_ is very high. The UK 6-month GLP-1 receptor agonist target is usually not reached.

## Introduction

The short-term aim of therapy for hyperglycaemia is improved blood glucose control without significant tolerability or safety issues, and with the longer-term objective of reducing vascular damage. Although initial pharmaceutical therapy is with an oral glucose-lowering agent, a steady decline in islet β-cell function results in progressive hyperglycaemia, which requires a stepwise escalation of treatment. Eventually insulin is often required as the only therapy independent of the need for endogenous insulin production.

Glucagon-like peptide-1 (GLP-1) receptor agonists have recently become a therapy option, with exenatide introduced into the UK market in 2007 and liraglutide in 2009. GLP-1 receptor agonists mimic, at supra-physiological levels, the action of endogenous GLP-1, in stimulating glucose-dependent insulin secretion and by suppressing glucagon secretion. Gastric emptying is delayed, especially in the early weeks of therapy. This, and perhaps a direct or indirect hypothalamic action, results in appetite/satiety changes and thus loss of body weight. In the UK, the National Institute for Health and Clinical Excellence (NICE) recommends a GLP-1 receptor agonist predominantly as a third-line treatment option in people who would otherwise start insulin and have a BMI ≥ 35.0 kg/m^2^ 1. NICE recommendations also state that therapy should be discontinued if the person has not had a beneficial metabolic response (a reduction of at least 11 mmol/mol (1.0%) in HbA_1c_ and weight loss of at least 3% of initial body weight at 6 months).

A review of 28 randomized clinical trials reported that GLP-1 receptor agonist therapy reduced HbA_1c_ by approximately 11 mmol/mol (1.0%), with weight loss of 2.3–5.5 kg 2. Comparisons with basal insulin have shown weight loss rather than gain with GLP-1 receptor agonists over 6 or 12 months and either no difference in HbA_1c_ reduction 3–5 or a greater decrease 6,7. Abstracts of observational studies in the USA and Germany have reported differences in populations who start a GLP-1 receptor agonist compared with those who initiate basal insulin therapy, and either no difference 8 or a greater reduction in HbA_1c_ on insulin 9.

The primary objective of the current study is to understand the characteristics of people beginning GLP-1 receptor agonist therapy in UK routine care, their metabolic response to treatment (glycaemic and weight control), which characteristics are associated with achieving glycaemic and weight control, and what percentage achieve the UK NICE targets. The characteristics and response to treatment were compared with those in people starting insulin therapy.

## Patients and methods

The study was an observational cohort study based on a database of UK primary care records.

### Source population

All data came from The Health Information Network (THIN), an observational database containing information collected in computerized primary care practices throughout the UK. Demographic and administrative data, primary care diagnoses and prescription treatments are routinely recorded against date in separate files within individual patient records. Details of referrals, secondary care diagnoses and deaths are also captured because of the structure of the UK health service. Major health events from before computerization are added retrospectively. Data on preventive practice may be recorded, including details of the annual diabetes review. Medical events are automatically coded at entry using the Read coding system 10. The source population comprised 4.3 million people permanently registered during the study period at the 429 practices on THIN which received laboratory results electronically. Approval for the study was given by the THIN Research Ethics Committee.

### Study population

The study population comprised of 4657 people with diabetes having at least 12 months of THIN records who were then prescribed a first-ever GLP-1 receptor agonist or basal insulin (detemir, glargine or NPH), from a baseline of two or three concomitant oral glucose-lowering agents, after 2006. The first GLP-1 receptor agonist or insulin prescription was the baseline treatment prescribed on the index date. Patients with no interpretable baseline HbA_1c_ in the prior 100 days or with more than one type of insulin/GLP-1 receptor agonist prescribed on this day were excluded. People with a record of any cancer (except non-melanoma skin cancer) or a prescription for a cancer therapy (British National Formulary category 8) or a glucocorticoid (British National Formulary category 6.3.2) in the year before index date were also excluded, as cancer or steroid-induced diabetes may require short-term glucose-lowering therapy 11.

### Baseline covariates

The following potential confounding variables were identified at baseline: age (1st July of the patient's year of birth), sex, year of index date, most recent body weight (at baseline or in the previous 6 months), duration of diabetes, BMI, estimated glomerular filtration rate (eGFR, if not recorded calculated from serum creatinine) and the most recent HbA_1c_. The duration of diabetes was estimated from the earliest record of a specific medical term, prescription of a glucose-lowering medication, an HbA_1c_ value or a diabetes health check. The number of oral glucose-lowering agents immediately pre-baseline was estimated from prescribing data in the previous year using a calculated modal dose for that form and strength if information on daily dose was not recorded. The number of oral glucose-lowering agents at baseline was defined as the number of oral glucose-lowering agents prescribed on the index date or in the next 100 days to allow for therapies that continued across the index date. A history of cardiovascular disease (myocardial infarction, angina, ischaemic heart disease, coronary heart disease, acute coronary syndrome or coronary revascularization), heart failure, microvascular complications (retinopathy, nephropathy or neuropathy) and severe gastrointestinal disease (pancreatitis, inflammatory bowel disease or diabetic gastroparesis) were identified from diagnoses recorded in the electronic clinical record.

### Statistical analysis

People were categorized into two primary cohorts by GLP-1 receptor agonist or insulin use at baseline. All were included as in an intention-to-treat type analysis, as later changes in therapy may be the result of limited efficacy of the first study treatment. For example, poor glycaemic control may result in an early switch in therapy. The primary cohorts were described and compared in terms of baseline covariates. Changing GLP-1 receptor agonist treatment or starting insulin treatment was identified for the GLP-1 receptor agonist cohort.

In those with sufficient follow-up, mean change from baseline in HbA_1c_ and weight in the reading nearest to 12 months after beginning study treatment (10–14 months window) was calculated. The association of treatment with mean change in HbA_1c_ and weight at 12 months was compared using a normal linear model adjusted by all baseline characteristics. Interaction tests with HbA_1c_ and weight change were completed for baseline age, weight, BMI, HbA_1c_, number of oral glucose-lowering agents prior to baseline and duration of diabetes to test for significance at the 1% level. A significant association was found between baseline HbA_1c_ and HbA_1c_ change at 12 months, so the adjusted comparison was repeated within quintiles of baseline HbA_1c_.

The association of baseline characteristics with decrease in HbA_1c_ and weight was examined by linear model in a univariate analyses. The baseline variables included were age, sex, BMI, weight, HbA_1c_, duration of diabetes, number of oral glucose-lowering agents pre- and post-treatment index date and a history of heart failure and cardio- or microvascular disease.

The change in weight and HbA_1c_ readings at 6 months (5–7 months window) were estimated for the GLP-1 receptor agonist cohort. The proportion who reached the NICE target [reduction of 3 kg in weight and 11 mmol/mol (1.0%) HbA_1c_] after 6 months on treatment were calculated. The percentage of the GLP-1 receptor agonist cohort who reached a weight reduction of >3 kg, and >6 kg by 12 months and mean weight change at 6 and 12months were also estimated. Data analysis used SAS software version 9.1 (SAS Institute, Cary, NC, USA).

## Results

### Treatments cohorts

The cohorts comprised 1123 people prescribed a GLP-1 receptor agonist and 1842 prescribed basal insulin after exclusions. The exclusions comprised 18.6% of the initial treated population who did not have an interpretable HbA_1c_ recorded in the 100 days before therapy [326 (16.7%) using a GLP-1 receptor agonist, 538 (19.9%) an insulin]. In addition, 216 (11.0%) prescribed a GLP-1 receptor agonist and 542 (20.4%) an insulin were excluded because of a cancer record, and 148 (7.6%) and 310 (11.5%), respectively, because of glucocorticoid use. Compared with insulin starters, those starting a GLP-1 receptor agonist had better glycaemic control and had higher BMI at baseline, and were younger with shorter duration of diabetes (Table 1). The GLP-1 receptor agonist users also had a larger number of oral glucose-lowering agents prescribed up to and when starting a new study regimen, and were less likely to have low GFR, microvascular disease and heart failure.

**Table 1:** Baseline characteristics of the primary cohorts

	GLP-1 receptor agonist mean (SD)	Insulin mean (SD)
Total, *n*	1123	1842
Age, years	56.2 (10.3)	63.4 (12.1)**
Diabetes duration, years	8.0 (4.5)	9.3 (6.4)**
Follow-up duration, years	1.1 (0.8)	1.9 (1.1)**
Male, *n* (%)	702 (62.5)	1127 (61.2)
Started therapy pre-2009, *n* (%)	193 (17.2)	1010 (54.8)**
Using two oral glucose-lowering agents up to baseline, *n* (%)†	711 (63.3)	1261 (68.5)*
Oral glucose-lowering agent at baseline, *n* (%):‡		
0	30 (2.7)	82 (4.5)
1	262 (23.3)	519 (28.2)**
2	660 (58.8)	1053 (57.2)
3	163 (14.5)	183 (9.9)
4	8 (0.7)	5 (0.3)
Weight, kg‡	111.5 (21.3)	88.5 (19.6)**
BMI, kg/m^2^	38.2 (6.7)	30.9 (6.3)**
HbA_1c_, mmol/mol (%)	78 (17)	84 (19)**
	9.3 (3.7)	9.8 (3.9)
eGFR <30 ml/min	3 (0.3)	30 (1.7)**
30-60 ml/min	120 (11.0)	400 (22.3)
>60 ml/min	972 (88.8)	1365 (76.0)
History, *n* (%) of:		
Gastrointestinal disease§	79 (7.0)	154 (8.4)
Cardiovascular disease¶	195 (17.4)	370 (20.1)
Microvascular disease††	304 (27.1)	619 (33.6)*
Heart failure	37 (3.3)	101 (5.5)*

* *P*<0.05,

** *P*<0.001.

† Rather than three agents.

‡ Percentage with missing data, GLP and insulin respectively: weight/BMI 8.4% and 11.1%; eGFR 2.5% and 2.6%.

§ Pancreatitis, inflammatory bowel disease or diabetic gastroparesis.

¶ Cardiovascular disease (myocardial infarction, angina, ischaemic heart disease, coronary heart disease, acute coronary syndrome or coronary revascularization).

†† Microvascular complications (retinopathy, nephropathy or neuropathy).

Mean study follow-up was shorter in the GLP-1 receptor agonist cohort because of starting therapy more recently—1.1 versus 1.9 years for insulin starters (Table 1). The majority (70%) of the total GLP-1 receptor agonist cohort were prescribed exenatide. The mean follow-up for liraglutide was 0.6 years compared with 1.4 years for exenatide, so the proportion of exenatide in the cohorts followed for 6-12 months will be higher. The majority of the insulin cohort had been prescribed insulin glargine (58%). A switch from first GLP-1 receptor agonist to the other occurred in 18.7% of the cohort during follow-up, while insulin was started in 23.7%. In the insulin cohort, 4.3% began a GLP-1 receptor agonist and 41.9% another insulin (switch or addition).

### Outcomes

The subgroups with at least 12 months’ post-treatment follow-up (intention-to-treat population) included 545 (48.5%) people from the GLP-1 receptor agonist cohort and 1340 (72.7%) from the insulin cohort. At 12 months, 366 (67.2%) of the GLP-1 receptor agonist cohort and 892 (66.6%) of the insulin cohort had an HbA_1c_ reported, and 335 (61.5%) and 634 (47.3%) had body weight data. The difference in baseline values for those who did or did not have a value at 12 months was not statistically significant for HbA_1c_ (*P*=0.09) or weight (*P*=0.6).

At 12 months, the mean unadjusted HbA_1c_ decrease from baseline was 7 mmol/mol (0.6%) in the GLP-1 receptor agonist cohort and 13 mmol/mol (1.2%) in the insulin cohort (Table 2). After adjustment, this difference was not statistically significant; adjusted mean difference −1.4 (95% CI 4.1, 1.2) mmol/mol. A test for interaction showed a clearly larger HbA_1c_ change at 12 months for the higher values of baseline HbA_1c_ with both therapies (*P*<0.001). In an adjusted comparison of quintile baseline HbA_1c_, the difference in HbA_1c_ reduction between basal insulin and GLP-1 receptor agonist was statistically significant only in those with baseline HbA_1c_ levels >96 mmol/mol (10.9%), adjusted mean difference −17.8 (95% CI 28.6, 7.0) (Table 3).

**Table 2:** Baseline and change in HbA_1c_ and body weight and comparison between the GLP-1 receptor agonist and insulin cohorts

Endpoint	GLP-1 receptor agonist mean (SD)	Insulin mean (SD)
HbA_1c_ (mmol/mol), *n* (%)*	366 (67.2)	892 (66.6)
Baseline	76 (16.4)	83 (18.5)
At 12 months	70 (18.9)	70 (16.9)
Change	−7 (17.6)	−13 (20.2)
Adjusted mean difference (95% CI)	—	−1.4 (−4.1, 1.2)
Body weight (kg), *n* (%)*	335 (61.5)	634 (47.3)
Baseline	112.1 (21.1)	90.0 (20.2)
12 months	107.6 (21.3)	91.5 (19.9)
Change	−4.5 (7.8)	+1.5 (5.4)
Adjusted mean difference (95% CI)	—	4.7 (3.7, 5.8)

* The number with reading at baseline and 12 months (% of those followed for 12 months), % units = mmol/mol × 0.0915 + 2.15. GLP-1 receptor agonist vs. insulin.

**Table 3:** Change of HbA_1c_ (mmol/mol)* by therapy according to quintiles of whole cohort baseline HbA_1c_

Quintile category	Baseline HbA_1c_		Mean HbA_1c_ change (sd)		Mean difference (95% CI)	
		
	GLP-1 receptor agonist mean (sd) *n*	Insulin mean (sd) *n*	GLP-1 receptor agonist	Insulin	Unadjusted	Adjusted
<66	58.5 (6.4) 108	61.6 (4.1) 152	1.7 (13.7)	0.2 (14.0)	−1.5 (−5.0, 1.9)	−1.8 (−6.6, 3.0)
66–75	70.6 (2.3) 80	70.8 (2.4) 202	−6.0 (14.5)	−6.1 (12.6)	−0.1 (−3.5, 3.4)	0.1 (−4.9, 5.1)
75–84	79.5 (2.7) 70	79.2 (2.5) 174	−7.6 (18.7)	−8.1 (15.5)	−0.5 (−5.1, 4.1)	1.3 (−4.6, 7.2)
84–96	89.3 (3.7) 63	89.7 (3.5) 190	−12.0 (19.4)	−17.3 (15.6)	−5.3 (−10.0, −0.5)	−5.4 (−11.5, 0.6)
>96	105.9 (10.3) 45	112.2 (15.1) 174	−17.4 (18.1)	−34.7 (22.9)	−17.3 (−24.5, −10.0)	−17.8 (−28.6, −7.0)

* Conversion to% units (mmol/mol × 0.0915) + 2.15 for absolute values, (mmol/mol × 0.0915) for change in value.

In univariate analyses of baseline characteristics, HbA_1c_ reduction was associated with baseline HbA_1c_ in both cohorts (*P*<0.001), as were increasing age in the GLP-1 receptor agonist cohort, and no microvascular disease or fewer oral glucose-lowering agents prescribed up to baseline in the insulin cohort (*P*<0.05 in each case). Other baseline variables were not associated with HbA_1c_ change. At 12 months, 30.3% of the GLP-1 receptor agonist cohort and 24.1% of the insulin cohort had an HbA_1c_ level below 58 mmol/mol (7.5%).

There was a significant difference in mean weight change at 12 months between cohorts, namely −4.5 kg after starting GLP-1 receptor agonist but +1.5 kg with insulin [adjusted mean difference 4.7 (95% CI 3.7, 5.8) kg] (Table 2). Weight change was highly variable, with a standard deviation of 7.8 kg with GLP-1 receptor agonist treatment and 5.4 kg with insulin. Mean weight reduction at 6 months was 4.2 kg on GLP-1 receptor agonist and 4.5 kg at 12 months, when 60.9% had lost more than >3 kg and 40.0% lost >6 kg. No statistically significant interactions were identified with weight change. Greater weight loss or lesser gain was seen with higher baseline body weight and BMI in both cohorts, although the association was stronger in the insulin cohort (*P*<0.001) for both characteristics compared with *P*<0.01 for higher weight and *P*<0.05 for BMI for the GLP-1 receptor agonist cohort. Greater weight loss or lesser gain was also found with lower baseline HbA_1c_ in both cohorts, and with oral glucose-lowering agent use at baseline with a GLP-1 receptor agonist (all *P*<0.01). No other baseline variables were associated with weight change.

The NICE targets are set for 6 months, when the GLP-1 receptor agonist cohort included 852 (75.9%) of the baseline sample, of whom 766 (89.9%) were prescribed a GLP-1 receptor agonist throughout; 229 (29.9%) of these people had both a body weight and HbA_1c_ record at 6 months. The NICE target of a combined reduction in HbA_1c_ of 11 mmol/mol (1.0%) and body weight of 3% was reached by 57 (24.9%) continuing treatment, 42.9% for HbA_1c_ and 59.5% for weight reduction individually, with 75.5% reaching at least one target.

## Discussion

This observational primary care study found large differences between the people who started GLP-1 receptor agonist therapy and those who started insulin after previous treatment with oral agents. In particular, the GLP-1 receptor agonist cohort had a much higher baseline mean weight and BMI and lower HbA_1c_. These differences are largely expected as the UK NICE clinical guideline indicates GLP-1 receptor agonist therapy mainly for those with BMI >35.0 kg/m^2^. The lower mean HbA_1c_ may account for the younger age and shorter duration of diabetes, and thus lower rates of microvascular disease and heart failure. The higher eGFR may be attributable to licensing restrictions for GLP-1 receptor agonists, and the finding that insulin is more commonly started in people with concomitant disease. The rate of gastrointestinal disease was similar between the treatment cohorts, despite some licensing restrictions in GLP-1 receptor agonist use, perhaps because pancreatitis, inflammatory bowel disease and gastroparesis are relatively uncommon.

Weight loss (rather than gain) has been confirmed by clinical trials 2 and observational studies^8^,^9^ of GLP-1 receptor agonists versus basal insulin. Our findings from routine care suggest that useful weight loss was obtained in people starting GLP-1 receptor agonists, although there was considerable variation. The insulin cohort gained weight only modestly. Those people with a higher baseline BMI benefited most from weight reduction whether treated with a GLP-1 receptor agonist or insulin. This was also true of lower HbA_1c_, as expected, given that reduction in urinary glucose excretion would be lower in this group.

The finding that a greater number of oral glucose-lowering agents at baseline decreased weight loss during GLP-1 receptor agonist treatment might be related to concomitant weight-promoting medications such as thiazolidinediones (and their cessation, which might itself reduce body weight). There was little further weight reduction between 6 months (4.2 kg) and 1 year (4.5 kg), consistent with the suggestion that a new level of calorie intake is established early in the course of therapy. A small US study of exenatide treatment reported that people had begun to regain weight by 1 year (−3.5 kg at 6 months, -2.1 kg at 12 months) 12.

Those people prescribed insulin had both a higher baseline HbA_1c_ and a greater mean reduction in HbA_1c_ compared with the GLP-1 receptor agonist cohort. However, the difference was not statistically significant in the adjusted analysis, and the reduction in HbA_1c_ was strongly associated with baseline HbA_1c_ regardless of treatment cohort (Table 3). In contrast, in those with very poor glucose control, HbA_1c_ reduction was substantially greater with insulin, perhaps reflecting more severe endogenous insulin deficiency in this group. The finding that HbA_1c_ reduction was negligible with either therapy in those who started treatment in the lowest HbA_1c_ quintile (<66 mmol/mol) is of concern, as these therapies have been shown to be effective at that level in the randomized trial setting. Perhaps the withdrawal of effective oral agents is not being overcome, or there remains a failure to titrate insulin doses to target over 6–12 months.

Clinical trials 3–5 and observational studies 8 of insulin versus exenatide have also reported no overall difference in change from a baseline HbA_1c_ after 6 or 12 months or a greater reduction in HbA_1c_ on exenatide 7 or liraglutide 6. One study reported a greater reduction in HbA_1c_ on insulin 9. Mean baseline HbA_1c_ in the clinical trials [57–71 mmol/mol (7.4–8.7%)] was lower than in our study and other observational studies [GLP-1 receptor agonist 63–78 mmol/mol (7.9–9.3%), insulin 77–84 mmol/mol (9.2–9.9%)], raising the issue of the generalizability of trial results. Those people with the highest HbA_1c_ who benefited most on insulin may have been excluded from clinical trials. The finding of a significantly greater drop in HbA_1c_ level on insulin rather than GLP-1 receptor agonist was reported from an observational study that had the greatest difference in baseline values 9. A problem of inadequate titration of insulin dose is clear in both settings and, judging by the mean 12-month level in this study, is a problem in routine UK practice.

Most people continuing GLP-1 receptor agonist treatment for 6 months did not reach the UK NICE target, 1 but many did benefit by reaching at least one element of the goal. However the determining factor for the NICE target, i.e. cost-effectiveness, was not usually met. Many people did not have values recorded around 6 months, suggesting the recommendations may not be followed.

This study used primary care records and, while this provided a picture of events in routine care, it resulted in a number of limitations. Almost one fifth of patients were excluded from the analysis because no interpretable HbA_1c_ was recorded in the 100 days prior to their first prescription for a study treatment. Some patients had no value recorded and it is possible that this group was treated in secondary care, biasing our results towards those monitored in primary care. Not all patients identified at first prescription had weight and HbA_1c_ readings at 6 or 12 months. However, there was little difference in the baseline weight and HbA_1c_ readings for the total cohorts and those subgroups analysed, although biased recording of later results cannot be ruled out. The results cannot be generalized to people who started treatment with other insulins or a mixture of GLP-1 receptor agonist and insulin, or to the unusual group who start insulin from no or one oral agent 13. It should also be noted that, while this study grouped patients regardless of insulin or GLP-1 receptor agonist type, differences in glycaemic control have been reported between agents. For example, while few patients had been treated with liraglutide for 12 months at the time of this study (11% of the GLP-1 receptor agonist cohort), the LEAD 6 study reported a significantly greater reduction in mean HbA_1c_ in liraglutide rather than exenatide treatment, but no difference in weight change 14.

The frequency of hypoglycaemia is also an important factor in determining the efficacy of diabetes therapies, but this endpoint was not studied because hypoglycaemia events managed by the patient, family or by paramedics are not well reported to the general practitioner and therefore often not included in the database. There may be bias in the reporting of events after insulin versus GLP-1 receptor agonist initiation. Clinical trials and other observational studies have reported similar 3,4,6 or lower overall rates of hypoglycaemia 5,8 or a lower rate of nocturnal events 3,4 in GLP-1 receptor agonist versus insulin treatment. Higher rates have also been reported in those with a history of hypoglycaemia 9.

In conclusion, GLP-1 receptor agonists and insulins are prescribed to different populations. In particular, GLP-1 receptor agonists are prescribed to those with higher BMI, in line with the benefits expected from clinical trial data, whereas those with higher HbA_1c_ are more likely to receive insulin. There was no sign of weight regain at 1 year. While mean weight loss was good (4.5 kg), the standard deviation of 7.8 kg implies a diverse response and many failed to reach a combined weight/HbA_1c_ target. A greater reduction in HbA_1c_ by the insulin cohort was clinically and statistically significant only in those with the highest baseline levels.

### Funding sources

The study was funded by Sanofi-Aventis without restriction on publication.

### Competing interests

GCH has received funding for research and payment for consultancy from a number of pharmaceutical companies, including manufacturers of insulin, GLP-1 receptor agonists and oral agents, and from charities, and has no direct stock holding in any pharmaceutical company. M-PD and EW are employees of Sanofi-Aventis. ADM has nothing to declare. Institutions associated with PH receive funding from many major pharmaceutical companies, including manufacturers of insulin, GLP-1 receptor agonists and oral agents.
